# Evaluating the latent structure of the non-social domain of autism in autistic adults

**DOI:** 10.1186/s13229-020-00401-x

**Published:** 2021-03-03

**Authors:** Rachel Grove, Sander Begeer, Anke M. Scheeren, Ricarda F. Weiland, Rosa A. Hoekstra

**Affiliations:** 1 Faculty of Health, University of Technology Sydney, Sydney, Australia; 2 Vrije Universiteit Amsterdam, Amsterdam, The Netherlands; 3 Institute of Psychiatry, Psychology and Neuroscience, King’s College London, London, UK

**Keywords:** Autism, Repetitive behaviours, Non-social autistic traits, Adults

## Abstract

**Background:**

The social domain of autism has been studied in depth, but the relationship between the non-social traits of autism has received less attention. The Diagnostic and Statistical Manual of Mental Disorders (DSM-5) outlines four criteria that make up the non-social domain including repetitive motor movements, insistence on sameness, restricted interests and sensory sensitivity. There is a lack of research into the relationship between these four criteria. This study aimed to evaluate the relationship between the non-social traits of autism in a large sample of autistic adults. It explored whether these traits are best conceptualised as four distinct factors, or exist along a single dimension.

**Methods:**

Participants included autistic adults from the Netherlands Autism Register. The four components identified within the DSM-5 non-social domain were measured by items from the Adult Routines Inventory, the Autism Spectrum Quotient short and the Sensory Perception Quotient short. Confirmatory factor analysis, as well as exploratory factor analysis and exploratory structural equation modelling, was implemented to examine the relationship between these four criteria.

**Results:**

Results indicated that a four-factor model provided the best fit, mapping onto the DSM-5 criteria. These four factors were moderately correlated, suggesting that four distinct, yet related factors best describe the non-social domain of autism. The one-factor model did not provide a good fit, highlighting that the non-social domain of autism is not a unitary construct.

**Limitations:**

The study included autistic adults who were cognitively able to complete the self-report measures. This may limit the generalisability of the findings to those who are less able to do so.

**Conclusions:**

This study provided evidence for the multidimensional nature of the non-social domain of autism. Given only two of the four criteria within the non-social domain need to be endorsed for a diagnosis of autism, there is room for substantial variation across individuals, who will have a unique profile within the non-social domain. The results have implications for our understanding of the heterogeneous nature of autistic traits, as well as for how we conceptualise autism as a diagnostic category. This is important for the provision of diagnosis and support within research and clinical practice.

## Background

The Diagnostic and Statistical Manual for Mental Disorders (DSM-5) [1] outlines two broad diagnostic domains for a diagnosis of autism spectrum disorder. These include difficulties in social communication and social interaction (Criterion A), as well as restricted, repetitive patterns of behaviour, interests, or activities (RRBI) (Criterion B) [1]. The non-social traits of autism, or RRBIs, are varied and can include special interests in particular topics, repetitive motor movements or speech, insistence on sameness or difficulty with changing routine [1]. In addition, the DSM-5 now also includes hypo- or hyper-sensory sensitivity under the RRBI domain [1]. The non-social domain of autism contains four arguably quite diverse criteria related to repetitive motor movements, insistence on sameness, restricted interests and sensory sensitivity. To add to the complexity, an individual only needs to meet two of the four components to endorse criterion B for a diagnosis. This means that there are 11 different combinations of RRBIs that can be met for a diagnosis of autism, resulting in the potential for substantial variability in symptom profiles and associated support needs.

The social and communication domain of autism has been studied in depth. Prior to the development of the DSM-5, social interaction and social communication were thought to represent two distinct diagnostic criteria for autism [2, 3]. However, given the large body of research outlining the relationship between these two constructs [4], they were combined in the DSM-5. There has been much research evaluating whether autism is best understood as a dimensional construct, existing along a continuum, or whether it represents a discrete category. This research has shown that social autistic traits can be conceptualised as falling along a continuum spanning across autistic individuals [5], as well as within the neurotypical population [6]. This dimensional assessment of autistic traits has provided better quantification of core autistic features [7]. However, while the social domain of autism has received a lot of attention, less has been given to the non-social domain, despite it also being a requirement for diagnosis. It is important to consider whether the non-social traits of autism should be conceptualised as a unitary or multidimensional construct.

Previous research has suggested that the non-social domain of autism may not represent a unitary construct. For example, a number of studies propose two subgroups of RRBIs in autistic children, including a repetitive motor and sensory behaviours factor, and an insistence on sameness factor [8–11]. Similarly, in neurotypical samples, other studies have also identified a two-factor structure comprised of motor behaviours or compulsions and rigidity or insistence on sameness [12, 13]. Further research has identified subgroups of autistic children containing differing levels of severity of RRBIs [14].

While there have been some attempts to understand the non-social domain of autism [8–11, 14], the majority of this research has been conducted with autistic children and adolescents. There has been one study that reported a similar two-factor model in a sample of autistic adults [15], comprising one repetitive sensory and motor behaviours factor and an insistence on sameness factor [15]. However, it is important to evaluate the non-social domain in more detail in autistic adults. The studies outlined above evaluated RRBIs using a single measure that did not provide adequate coverage of the four RRBI criteria. The majority of these studies used the Autism Diagnostic Interview Schedule—Revised (ADI-R) [16], which contains only 12 items relating to RRBIs. Other research has used more comprehensive measures of RRBIs, such as the Repetitive Behavior Scale—Revised (RBS-R) [17]. The RBS-R is a parent report measure that contains 43 items evaluating RRBIs, including stereotyped, self-injurious, compulsive, ritualistic, restricted and sameness behaviours [17]. This parent report measure has been shown to be a valid dimensional measure of RRBIs in autistic children [18, 19]. Additional research has used self-report measures in adults, including the Adult Repetitive Behaviour Questionnaire (RBQ-2A) [20] and Adult Routines Inventory (ARI) [12]. The RBQ-2A contains 20 items evaluating RRBIs, three of which assess sensory sensitivity. The ARI is a more comprehensive measure, with 55 items evaluating RRBIs, and also containing a small number of items assessing sensory issues. In addition, there have been some attempts to capture the new sensory sensitivity criterion using dimensional measures such as the Short Sensory Profile [21] for children, as well as the Sensory Perception Quotient [22] for adults. However, no previous studies have simultaneously included comprehensive coverage of all these four components of the DSM-5 non-social domain of autism, including repetitive motor movements, insistence on sameness, restricted interests and sensory sensitivity.

The evidence for the non-social domain as a unitary or multidimensional construct is limited, as research into the association between the four RRBI criteria is lacking. There is a need for an in-depth examination of the relationship between the four components of the DSM-5 RRBI criterion. This study aims to evaluate the relationship between the non-social traits of autism in a large sample of autistic adults. It will determine whether the non-social domain is best conceptualised by four distinct factors, as outlined in the DSM-5, or whether these RRBIs exist along either a single dimension or fit a two-factor structure, as identified by previous research.

## Methods

### Participants

Participants were recruited through the Netherlands Autism Register (NAR), a register of research volunteers of autistic children and adults. The total sample consisted of 833 autistic adults (478 females and 355 males). The sample was restricted to participants who reported that they had received an official diagnosis of an autism spectrum disorder using the DSM-IV [2] or DSM-5 [1] criteria. In addition, this diagnosis was required to be provided by a qualified health professional. The majority of the sample indicated that they were diagnosed by a psychologist (67%) or psychiatrist (27%). The mean age of the sample was 44.7 years (sd 13.6). The mean age of diagnosis was 36.5 years (sd = 15.0). The relatively late age of diagnosis is in line with other studies that have included adult participants up to old age [23, 24]. A proportion of the sample (N = 406) completed the Raven's Progressive Matrices Clinical Edition (Raven's 2) [25], that provides and online IQ score and percentile rank. The majority of the sample had IQ scores above 86. Further details regarding the sample composition are provided in Table 1. Additional details regarding the NAR can be obtained from www.nederlandsautismeregister.nl/english/.

### Measures

#### Adult routines inventory

The Adult Routines Inventory (ARI) [12] is a 55-item measure of restricted and repetitive behaviours and interests. Items are scored on a five-point Likert scale from 1 ‘not at all/never’ to 5 ‘very much/always’. In the original publication, the ARI was evaluated in a general population sample of 3,108 adults (966 men) [12]. In this non-clinical sample, a two-factor structure was reported with one ‘motor behaviours or compulsions’ subscale and one ‘rigidity or insistence on sameness’ subscale. Higher scores indicate a higher level of restricted and repetitive behaviours and interests. The ARI showed excellent internal consistency in the current study sample across each subscale (motor behaviours or compulsions subscale: Cronbach’s alpha = 0.87; rigidity or insistence on sameness subscale: Cronbach’s alpha = 0.92).

#### Sensory perception quotient

The short version of the Sensory Perception Quotient (SPQ-short) [22] contains 35 items and assesses hypo- and hyper-sensory sensitivity across all five senses. Items are scored on a four-point scale from 0 ‘strongly agree’ to 3 ‘strongly disagree’. Items are summed to create a total score. Higher scores indicate less sensory sensitivity. To ease interpretation of the data, the SPQ-short was reversed scored in the current study so that higher scores indicated more sensory sensitivity. The factor structure of the Dutch SPQ-short has previously been evaluated in the NAR data, outlining a hierarchical model containing a general sensory sensitivity factor, with five subfactors across the five modalities of vision, taste, hearing, smell and touch [26]. The fit of this hierarchical model of the SPQ-short was evaluated in the current sample (which overlapped 55% with the previous publication), providing an adequate fit to the data (RMSEA = 0.06, CFI = 0.91, TLI = 0.90). The SPQ-short had good internal consistency within the current sample (Cronbach’s alpha = 0.89).

#### Autism spectrum quotient

The short form of the Autism Spectrum Quotient (AQ-short) [27] is a 28-item measure that assesses autistic traits. The AQ-short consists of a higher-order ‘Social Behaviour’ factor, measuring social skills, imagination, routine and switching, as well as a ‘Numbers and Patterns’ factor, which focuses specifically on an interest in numbers, dates, patterns and categories of things. This two-factor structure was replicated in a subsequent factor analysis in the NAR [28], as well as confirmed within the current sample (RMSEA = 0.06, CFI = 0.92, TLI = 0.91), of which the sample had 62% overlap with the previously reported paper. As this paper focuses specifically on RRBIs, the Social Behaviour factor was excluded, and only the Numbers and Patterns subscale was included in the current analyses. Higher scores on this subscale indicate a greater interest in numbers and patterns. The AQ-short has good internal consistency and test–retest reliability [29]. The numbers and patterns subscale displayed good reliability in this study sample (Cronbach’s alpha = 0.90).

### Statistical analyses

The factor structure of the SPQ-short and AQ-short has been assessed in both general and autistic populations [22, 29] and has previously been evaluated in the NAR data [26, 28] and the current sample. In contrast, the factor structure of the ARI [12] is based on a general population sample only. We therefore started our analyses with examining the factor structure of the ARI in our autistic sample.

#### Factor structure of the ARI

Confirmatory factor analysis (CFA), as well as exploratory factor analysis (EFA) and exploratory structural equation modelling (ESEM), was implemented to examine whether the previously reported ARI structure also applied to our sample of autistic participants. The initial step involved implementing a CFA model to test the fit of the two-factor structure outlined by the authors [12]. The CFA model was implemented using the weighted least square mean and variance-adjusted (WLSMV) estimator with categorical indicators. All factors were allowed to correlate within this model. Following the CFA model, EFA was implemented to explore the fit of the ARI to the current data. The EFA models were implemented using the WLSMV estimator, using the oblique geomin rotation procedure. This rotation method estimates the factor intercepts and residual variances. This rotation method allows the factors to be correlated, with the variance of each factor estimated at 1 [30]. ESEM has been shown to provide an alternative to confirmatory models, where it is a requirement that there are zero cross-loadings across variables [30]. ESEM combines both confirmatory and exploratory procedures [30]. While the number of factors is provided within the model (as in CFA), each item is allowed to cross-load across each factor (similar to EFA methods). This is a strength of the ESEM approach and is particularly useful when a measure contains a number of related items assessing a particular construct [30], such as the ARI. ESEM was implemented following the CFA and EFA models to evaluate the fit of the ARI within this sample, allowing the items to load across each factor. Similar to the EFA models, the ESEM models used the WLSMV estimator, and factors correlations were allowed.

The CFA, EFA and ESEM models were evaluated based on a number of fit indices, including the Bayesian information criterion (BIC) [31], Akaike information criterion (AIC) [32], sample size-adjusted BIC (SSABIC) [33], root mean square error of approximation (RMSEA) [34], comparative fit index (CFI) [35] and Tucker–Lewis index (TLI) [36]. Smaller BIC, AIC and SSABIC values indicate a better model fit. CFI and TLI values > = 0.95 indicate very good fit of the model, with values > = 0.90 indicating adequate fit [37, 38]. RMSEA values < = 0.08 indicate a good fitting model, with values < = 0.05 indicating excellent fit to the data [39]. The decision to remove items followed the recommendations outlined by Costello and Osborne [40]. The authors recommend removing items that contain factor loadings < 0.32 from the model, as well as items containing cross-loadings across multiple factors [40].

#### DSM-5 non-social domain of autism

**Fig. 1:**
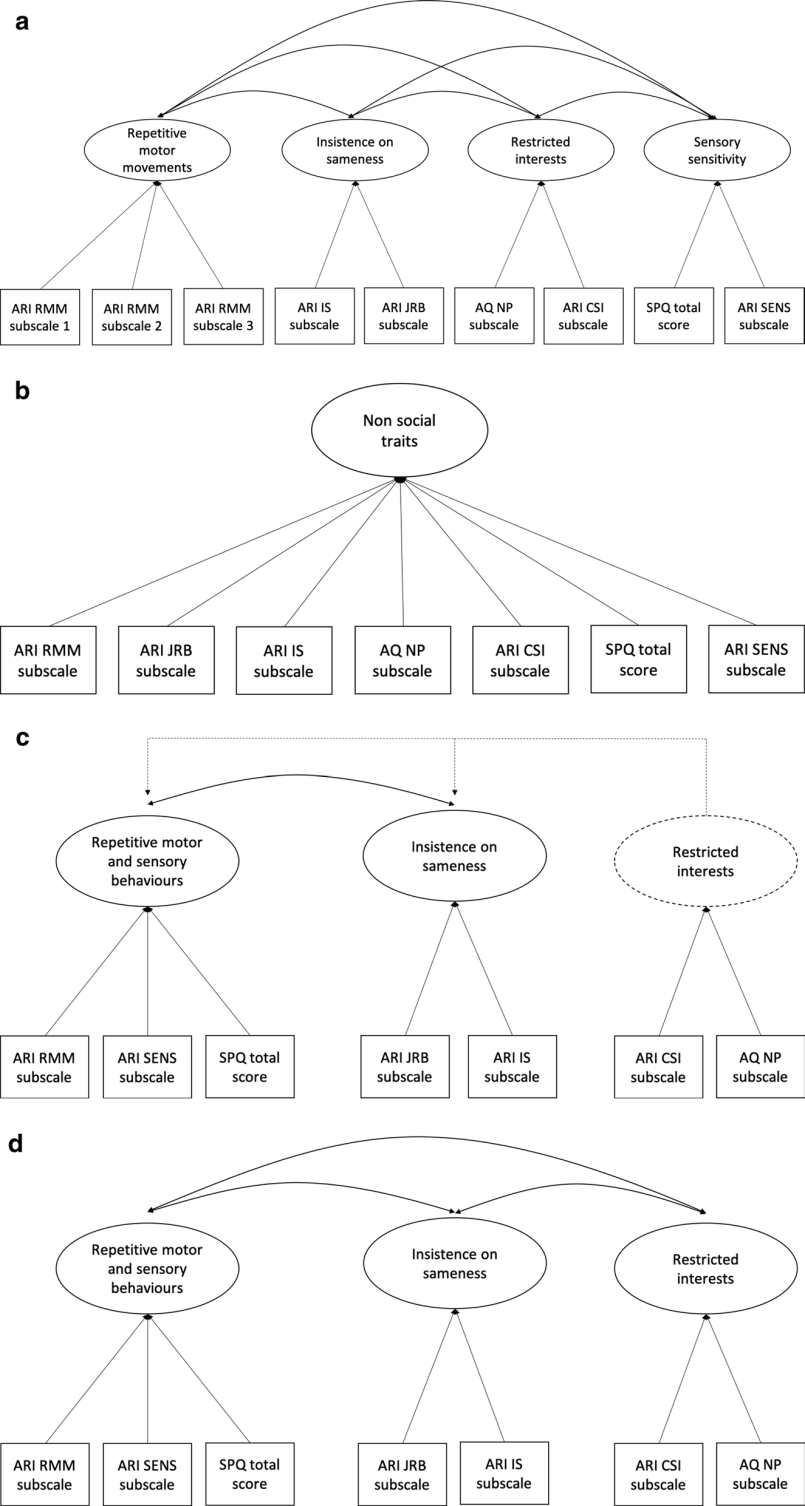
Potential models of non-social autistic traits. **a.** Four-factor multidimensional DSM-5 model. **b.** One-factor dimensional model. **c.** Two-factor model. *Note* Unclear whether restricted interests should be combined with factor 1 or 2. **d** Three-factor model. *Note* ARI = adult routines inventory; RMM = repetitive motor movements; SENS = sensory sensitivity; JRB = just right behaviours; IS = insistence on sameness; CSI = compulsions/special interests; SPQ = sensory perception quotient; AQ = autism spectrum quotient; NP = numbers and patterns

The DSM-5 outlines four criteria under the non-social domain. These include repetitive motor movements, insistence on sameness, restricted interests and sensory sensitivity [1]. There are a number of potential relationships between these four components (Fig. 1). This study implemented a number of different models to evaluate the relationships outlined in Fig. 1. A four-factor CFA model was fit to the data (Model 1), including four distinct but related factors as outlined in the DSM-5 (four-factor model). Four additional CFA models were also implemented to evaluate the alternative outcomes. A one-factor model was included to assess whether the four criteria outlined in the DSM-5 exist along a single continuum (Model 2). Two additional models were implemented to evaluate the two-factor model commonly identified by previous research, which includes a repetitive motor and sensory behaviours (RMS) factor and an insistence on sameness (IS) factor. Given that these previous models do not include restricted interests, it was unclear as to where this criterion would load. To explore which model fit the data best, we tested two alternative models. Model 3 allowed restricted interests to load onto the RMS factor, while Model 4 included restricted interests with the IS factor. A final three-factor model including an RMS and IS factor, as well as a separate restricted interests factor, was also fit to the data (Model 5).

Model 1 includes a repetitive motor movements factor, corresponding to the repetitive motor movements subscale of the ARI. However, in order to be identified, each factor within a CFA model requires two or more variables [41]. In order to account for this, the items within the repetitive motor movements subscale of the ARI were parcelled into three subscales, two containing five items and one with four items. These were parcelled using the random parcelling technique [42]. This method has been recommended by previous research within unidimensional indicators [43, 44]. This also enabled all models within the analyses of the non-social domain to be directly compared using the maximum likelihood estimation with robust standard errors (MLR) indicator. Some authors argue that each latent variable within a CFA model requires three or more indicators in order to be identified [45]. However, Kenny and Milan [41] state that it is appropriate to use two indicators per latent variable if each indicator loads onto one latent variable, there are no correlated errors, and there are at least two correlated latent variables within the CFA model. Given these parameters were met within the current sample, two indicator variables were utilised for the factors evaluating insistence on sameness, restricted interests and sensory sensitivity within Models 1 and 5.

The non-social models were evaluated using the above fit criteria. All variables within Models 1 to 5 were standardised prior to analysis, resulting in a mean of zero and a variance of one. This enabled the comparison of multiple measures that were assessed on different scales. In addition, to identify each latent variable within the CFA model in Mplus the variance of each factor was fixed at 1 [30]. All analyses, as well as the item standardisation, were conducted in Mplus, version 8.3 [46].

## Results

An outline of the sample characteristics, as well as mean scores across all measures, is provided in Table 1.

**Table 1:** Characteristics of the sample

Sex	N	%
Females	478	57.4
Males	355	42.6

Raven’s IQ score		
Above 130	37	9.1
116 to 130	84	20.7
86 to 115	244	60.1
71 to 85	32	7.9
56 to 70	9	2.2

Age and diagnosis	N	Mean (SD)	Range
Age	833	44.7 (13.6)	16–82
Age of diagnosis	760	36.5 (15.0)	3–75
Time since diagnosis (years)	760	8.3 (5.6)	0.01– 31.9

Measures			
*Adult Routines Index*			
Insistence on sameness	833	41.6 (8.9)	12–60
Repetitive motor behaviours	833	31.7 (10.1)	14–64
Just right behaviours	833	20.3 (6.2)	7–35
Sensory sensitivity	833	20.5 (5.4)	6–30
Compulsions/special interests	833	12.3 (3.9)	5–25
*Sensory Perception Quotient short*			
Total score	462	60.1 (15.4)	12–102
*Autism Spectrum Quotient short*			
Numbers and patterns	793	13.7 (3.9)	5–20

### ARI analyses

Results from the ARI analyses are provided in Table 2. The two-factor CFA model did not provide a good fit to the data. EFA models indicated that a five-factor model provided the best fit. These factors included items evaluating insistence on sameness, just right behaviours, repetitive motor behaviours, sensory sensitivity and compulsions/special interests. Across the EFA models, there were five items that did not load onto any of the five factors identified. These included item 12 (Are you a picky eater?), item 14 (Do you enjoy collecting things?), item 15 (Do you focus on details when doing a task?), item 33 (Do you crack your joints (knuckles, neck, back, jaw, etc.)?) and item 39 (Do you like to have a sense of evenness or balance, so if something touches one side of your body you have the urge to have it touch the other side of your body?). These items did not appear to measure a separate construct, but were random items that did not load onto any of the factors identified (Additional file 1: Table 1). These items were therefore dropped from the subsequent ESEM analysis. In addition, there were six items that contained significant cross-loadings across factors. Item 17 (Do you notice imperfections in objects, like scratches on furniture, spots/stains, or frays on clothing, etc.?), item 18 (Do you prefer to finish one task before moving on to the next?), item 27 (Do you notice when pictures on walls are not lined up, or are crooked?), item 28 (Do you feel you have to complete a task once you have started it?), item 45 (Do you like to go to new places?) and item 53 (Do you like to try new things?) were also excluded based on the recommendations outlined by previous research [40]. This final ESEM comprising all remaining 44 items contained a RMSEA < 0.05, indicating an excellent fit to the data. The CFI was also above 0.95, indicating an excellent fit. The TFI was above the recommended threshold of 0.90, indicating a good fit of this model to the data. The items loading onto each factor are outlined in Additional file 1: Table 2.

**Table 2:** Fit indices and model comparisons of the Adult Routines Inventory

Model	Description	Fit indices				
		RMSEA	CFI	TLI	χ^2^ (df)	No. of free parameters
Evans et al. [12] two-factor model						
1	Two-factor CFA model (n = 833)	0.073	0.858	0.851	4712.245** (859)	216
EFA model						
2	Four-factor EFA model (n = 833)	0.056	0.907	0.891	4536.899** (1271)	214
3	Five-factor EFA model (n = 833)	0.050	0.926	0.910	3792.204** (1220)	265
ESEM model						
4	Five-factor ESEM (n = 833) including 44 items	0.047	0.956	0.943	2065.353** (736)	386

*Note* RMSEA, root mean square error of approximation; CFI, comparative fit index; TLI, Tucker–Lewis index; χ^2^, Chi-square statistic; df, degrees of freedom

** *p* < 0.01

Subsequent factor analyses exploring the dimensionality of the DSM-5 non-social domain implemented these five ARI subscales. The AQ-short numbers and patterns subscale and the SPQ-short were also included in the factor models. Within the four-factor DSM-5 model (Model 1), the three item parcels from the ARI repetitive movements subscale were included in the model rather than the total subscale score. This was to ensure that the model was identified. The descriptive statistics of the five ARI subscales are provided in Table 1. Correlations between the ARI subscales and the SPQ-short and AQ-short are provided in Table 3.

**Table 3:** Correlation between the Adult Routines Inventory, Sensory Perception Quotient short and the Autism Spectrum Quotient short

	ARI IS	ARI JRB	ARI MOT	ARI SENS	ARI CSI	SPQ	AQ NP
ARI IS	1						
ARI JRB	0.69**	1					
ARI MOT	0.43**	0.37**	1				
ARI SENS	0.48**	0.42**	0.49**	1			
ARI CSI	0.47**	0.39**	0.44**	0.37**	1		
SPQ	0.33**	0.35**	0.35**	0.60**	0.34**	1	
AQ NP	0.35**	0.32**	0.32**	0.29**	0.40**	0.33**	1

ARI IS = Adult Routines Inventory insistence on sameness subscale; ARI MOT = Adult Routines Inventory routine repetitive motor behaviours subscale; ARI JRB = Adult Routines Inventory just right behaviours subscale; ARI SENS = Adult Routines Inventory sensory sensitivity subscale; ARI CSI = Adult Routines Inventory compulsions/special interests subscale; SPQ = Sensory Perception Quotient short; AQ NP = Autism Spectrum Quotient short numbers and patterns subscale

** *p* < 0.01

### DSM-5 non-social domain

Results from the CFA models evaluating the RRBI domain indicated that the four-factor model (Model 1) provided an excellent fit to the data (Table 4). The one-factor CFA model (Model 2) evaluating whether the non-social traits of autism fall along the same unitary dimension did not provide a good fit to the data (RMSEA = 0.19, CFI = 0.74, TLI = 0.63). The additional models (Models 3 to 5) evaluating the RMS, IS and restricted interests factors had CFI, TLI and RMSEA values outside the recommended thresholds, indicating that they did not provide a good fit. Overall, fit indices indicated the four-factor model described the data best.

**Table 4:** Fit indices and model comparisons of the non-social traits of autism (Adult Routines Inventory, Sensory Perception Quotient short, Autism Spectrum Quotient short)

Model		Description	Fit indices			
			RMSEA	CFI	TLI	χ^2^
Non-social model						
1		Four-factor DSM-5 model	0.031	0.994	0.989	37.943**
2		One-factor non-social model	0.139	0.858	0.786	238.902**
3		Two-factor RMS and IS model A (restricted interests loading onto RMS factor)	0.090	0.944	0.910	100.910**
4		Two-factor RMS and IS model B (restricted interests loading onto IS factor)	0.107	0.921	0.872	137.767**
5		Three-factor model RMS, IS and restricted interests (RI) loading onto a separate factor	0.077	0.965	0.934	65.610**

Note. RMSEA, root mean square error of approximation; CFI, comparative fit index; TLI, Tucker–Lewis index; χ^2^, Chi-square statistic

** *p* < 0.01

**Fig. 2:**
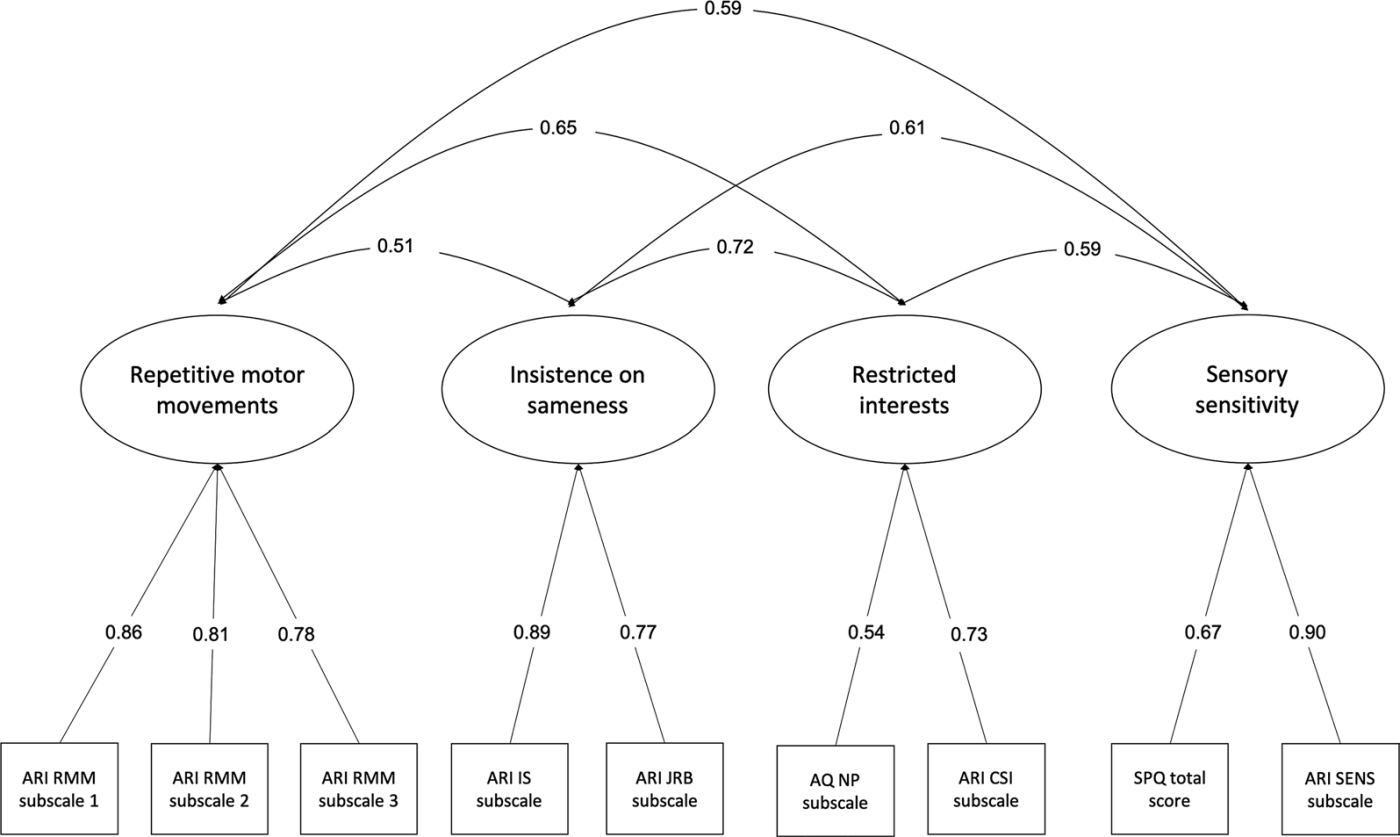
DSM-5 four-factor CFA model of non-social autistic traits

The four-factor DSM-5 model is given in Fig. 2. The four factors measuring repetitive motor movements, insistence on sameness, restricted interests and sensory sensitivity were moderately correlated with each other (Table 5).

## Discussion

This study aimed to evaluate the relationship between the non-social traits of autism in a large sample of autistic adults. In the process, it also evaluated the factor structure of the ARI questionnaire in a sample of autistic adults. The results indicated that the ARI has a five-factor structure in this autistic population. This is different to the two-factor model identified in a non-clinical sample by the original study [12]. It may be that the ARI functions differently for autistic adults compared with a neurotypical sample. Future research should replicate these analyses with more samples of autistic adults in order to clarify the factor structure of the ARI.

The results indicated that a four-factor DSM-5 model provided the best fit. These four continuous factors or dimensions mapped onto repetitive motor movements, insistence on sameness, restricted interests and sensory sensitivity. This provides evidence that the non-social domain of autism is multidimensional, consisting of four factors rather than one or two broader factors. These factors were shown to be moderately correlated with each other, suggesting four distinct, yet related factors of RRBIs in autistic adults.

The results of the current study were in contrast to previous research that outlines two dimensions underlying RRBIs in both autistic children, adults and neurotypical samples, including a factor consisting of repetitive motor and sensory behaviours, and a factor measuring rigidity or insistence on sameness [8–13, 15]. These previous studies utilised only one measure of RRBIs, while the current study used three measures to assess RRBIs in depth, including the ARI [12], the SPQ-short [22] and the AQ-short [27]. Future research would benefit from similar comprehensive coverage of all RRBI criteria. This is also important when considering a diagnostic assessment of autism, to ensure that all these domains are covered sufficiently. The differing results across studies also highlight the importance of evaluating RRBIs within samples of autistic adults, rather than just samples of children and adolescents.

While there was evidence for four distinct dimensions characterising RRBIs in the current study, these factors were correlated. The largest correlation was observed between restricted interests and insistence on sameness. This is consistent with previous research outlining a relationship between cognitive control related to restricted interests and insistence on sameness [47]. There was also a moderate correlation between sensory sensitivity and repetitive motor movements. This is somewhat consistent with previous research that includes repetitive motor movements and sensory behaviours within the same subgroup of RRBIs [8–11]. However, an alternative explanation for the correlation is that some of the items within these factors were derived from the same questionnaire (i.e. the ARI), which may have resulted in inflated item associations. Sensory sensitivity was also moderately correlated with insistence on sameness. There has been some suggestion that there is a relationship between insistence on sameness and sensory behaviours [48]. It has been proposed that RRBIs may serve to compensate for over or under sensory arousal and that this may be related to anxiety [48]. Further research evaluating the nature of this relationship between RRBIs and anxiety in autistic adults is needed. Sensory sensitivity was moderately correlated with restricted interests, highlighting a relationship between this construct and the other three RRBI criteria. This is consistent with previous research outlining a relationship between sensory sensitivity and RRBIs [49]. This also indicates that sensory sensitivity forms an important part of the non-social traits of autism.

The results suggest that a one-factor model did not provide a good fit to the data. This indicates that the non-social domain of autism is not a unitary construct. This is interesting, as the majority of the research suggests that the social communication domain of autism falls along a single quantitative dimension spanning into the general population [6, 50]. There have only been a limited number of studies that evaluate RRBIs in relatives of autistic individuals. This research has provided some evidence for a broader autism phenotype in this non-social domain, including the presence of broad stereotyped behaviours and rigid personality type [51]. However, more research is needed to evaluate the nature of RRBIs within the general population and whether this domain is similarly multifactorial in non-clinical samples.

**Fig. 3:**
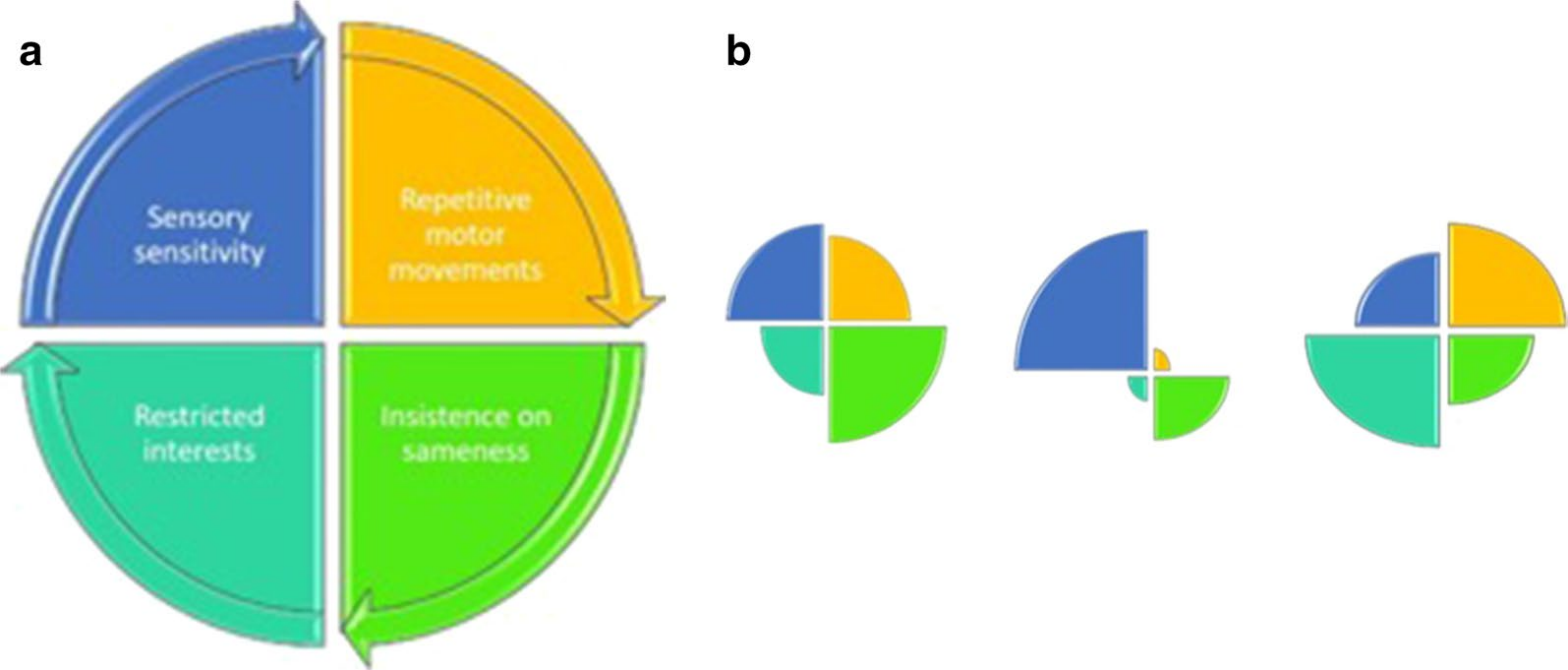
**a** Multidimensional model of non-social autistic traits. **b** Three examples of possible non-social symptom profiles of autistic individuals. *Note* Adapted from United States Government Accountability Office (2016) report Fig. 2

The DSM-5 diagnostic criteria outline that only two out of four RRBI criteria need to be met to qualify for a diagnosis of autism. By definition, this means there is substantial variation in the frequency and patterns of symptom endorsement (Fig. 3). Figure 3b outlines some examples of potential unique profiles of endorsement of the four RRBI criteria. This results in large variation in non-social autistic traits in clinical samples. This has implications for research, as what we observe autism to ‘be’ depends on how we define what autism ‘is’. This may explain part of the variation in findings across genetic studies, as well as in outcome studies of support programs [52, 53]. The generalisability of results across clinical samples is also problematic given this potential variation. While this is beyond the scope of the current study, there is a need to determine the differential impact of endorsing each of the four RRBI criteria, as well as the implications this has on diagnosis, clinical practice and outcomes for autistic individuals.

The multidimensional nature of RRBIs signifies an added layer of heterogeneity in autism that is important to understand. Combining findings from previous studies that suggest a quantitative nature of both the social and non-social domains [6, 50, 51], with the results presented here, autism appears best conceptualised under two broad social and non-social dimensions, with four second-order dimensions of RRBIs. This latent structure of the autism phenotype allows for unique individual symptom profiles that may vary considerably across each dimension. If this latent structure is replicated in future studies, we should consider how best to delineate thresholds to distinguish those individuals who require a diagnosis and those who have sub-clinical levels of autistic traits.

The findings reported here raise questions pertinent to our understanding of the nosology or definition of autism. Given the inherent heterogeneity in autism, it has been suggested that it may be more appropriate to conceptualise autism as ‘the autisms’, rather than as a unitary disorder [54]. Relevant to this is the notion of ‘lumpers’ and ‘splitters’ among researchers. Splitters would argue that it is important to define a number of separate unique conditions, whereas lumpers would argue for condensing categories to combine similar constructs [55]. Previous research has provided support for the lumpers and the DSM-5 (which amalgamated autism subtypes as described in the DSM-IV into one category ‘Autism Spectrum Disorder’). This highlights that the DSM-IV autism subtype diagnoses were unreliable [56]. Further support for the DSM-5 model of autism, which specifies autism according to two broad domains (social communication and RRBI), rather than the three domains included in the DSM-IV, has been provided by a number of authors [4, 57]. This is also reflected in the latest release of the International Classification of Diseases (ICD-11) [58], which includes two broad domains of social interaction and communication and restricted, repetitive and inflexible patterns of behaviour and interests. While the DSM-5 is used predominantly in the USA, the ICD-11 is used worldwide. Therefore, the global consensus on the diagnosis of autism has narrowed significantly.

The current study contributes to these findings, indicating that the non-social domain of autism is multidimensional and fits with the definitions outlined in the DSM-5 and ICD-11. The four factors were moderately correlated, suggesting that these are distinct. This indicates that, while the lumping position has worked well overall, if we want to better understand autism heterogeneity, it may be important to evaluate the utility of splitting the four factors of the RRBI and considering these symptom profiles separately. This is consistent with previous research that argues that the dimensional approach taken by the DSM-5 provides an opportunity to identify subtypes of autism [59]. However, it is important to consider the added value of creating subtypes of autism based on RRBIs. The differentiation between subgroups would need to be made reliably. In addition, the clinical utility of these subgroups would also need to be considered. More research is needed to establish whether RRBI subtypes exist and whether these subtypes may be associated with different support needs or trajectories over time.

## Limitations

The voluntary online nature of the data collection in this study meant that it was not possible to confirm clinical diagnoses in the entire sample. However, previous research has shown that diagnoses reported via online registers are reliable [60]. Stringent inclusion criteria were also applied to the sample in order to ensure that all reported diagnoses had been provided by qualified practitioners based on the DSM-IV or DSM-5 criteria. There is an issue with circularity in this study, as the participants in this study (who all have a formal clinical autism diagnosis) inherently endorse at least two out of four of the RRBI criteria. In future studies, it would be good to include people who have not received a diagnosis, but may have subthreshold levels of autistic traits. This would assist with determining the relationship between these factors in samples with varying levels of autistic traits. The data also included self-report measures, so are limited to those who are cognitively able to complete these assessments. This may therefore limit the generalisability of the findings to individuals who may be less able to do so.

## Conclusions

This study evaluated the structure of the non-social domain of autism. It found evidence for a multidimensional model mapping onto the DSM-5 criteria including repetitive motor movements, insistence on sameness, restricted interests and sensory sensitivity. These criteria were moderately correlated with each other, indicating four distinct, yet related factors. This research provides evidence for the importance of including multiple measures to provide an in-depth evaluation of RRBIs in autistic adults. The results have implications for our understanding of the nature of autistic traits, as well as for how we conceptualise autism as a diagnostic category. It is important to recognise that there are limits within the findings relating to nosological refinement, given the inherent complexities and heterogeneity of autism, as well as the difference in findings across child and adult samples. However, the search for the refinement of our definitions of autism is vital for autistic individuals and their families, as well as service providers, researchers and practitioners, as it has a significant impact on the provision of diagnosis and support within research and clinical practice.

**Table 5:** Correlations between the four DSM-5 non-social factors

	Repetitive motor movements	Insistence on sameness	Restricted interests	Sensory sensitivity
Repetitive motor movements	1			
Insistence on sameness	0.51**	1		
Restricted interests	0.65**	0.72**	1	
Sensory sensitivity	0.59**	0.61**	0.59**	1

** *p* < 0.01

## Supplementary information

**Additional file 1**. **Table 1**: Excluded item loadings from the five-factor EFA of the Adult Routines Inventory. **Table 2**: Item distribution of the Adult Routines Inventory subscales from the ESEM.

## Data Availability

The data that support the findings of this study are available from the Netherlands Autism Register (https://www.nederlandsautismeregister.nl/english/), but restrictions apply to the availability of these data, which were used under licence for the current study, and so are not publicly available.

## Abbreviations

RBQ-2A: Adult Repetitive Behaviour Questionnaire
ARI: Adult Routines Inventory
AIC: Akaike information criterion
ADI-R: Autism Diagnostic Interview Schedule—revised
AQ-short: Autism Spectrum Quotient short
BIC: Bayesian information criterion
CFI: Comparative fit index
CFA: Confirmatory factor analysis
DSM-5: Diagnostic and Statistical Manual of Mental Disorders—fifth edition
DSM-IV: Diagnostic and Statistical Manual of Mental Disorders—fourth edition
EFA: Exploratory factor analysis
ESEM: Exploratory structural equation modelling
IS: Insistence on sameness
MLR: Maximum likelihood estimation with robust standard errors
NAR: Netherlands Autism Register
RBS-R: Repetitive Behavior Scale-Revised
RMS: Repetitive motor and sensory behaviours
RRBI: Restricted, repetitive patterns of behaviour, interests, or activities
RMSEA: Root mean square error of approximation
SSABIC: Sample size-adjusted Bayesian information criterion
SPQ-short: Sensory Perception Quotient short
TLI: Tucker–Lewis index
WLSMV: Weighted least square mean and variance adjusted estimation
