# Supplementary material for: Evaluating the latent structure of the non-social domain of autism in autistic adults
Source: Mol Autism. 2021 Mar 3;12:22. doi: 10.1186/s13229-020-00401-x (PMC7931608; doi:10.1186/s13229-020-00401-x)
Supplement: Supplementary file 1 — Additional file 1. Table 1: Excluded item loadings from the five-factor EFA of the Adult Routines Inventory. Table 2: Item distribution of the Adult Routines Inventory subscales from the ESEM. [file 13229_2020_401_MOESM1_ESM.docx]

| **Supplementary Table 1** Excluded item loadings from the 5 factor EFA of the Adult Routines Inventory | | | | | |
| --- | --- | --- | --- | --- | --- |
|  | **Factor loadings** | | | | |
| **Item** | **F1** | **F2** | **F3** | **F4** | **F5** |
| 12. Are you a picky eater? | 0.16 | 0.10 | 0.28 | 0.28 | -0.10 |
| 14. Do you enjoy collecting things? | 0.09 | 0.06 | 0.25 | -0.03 | 0.26 |
| 15. Do you focus on details when doing a task? | 0.21 | 0.19 | 0.07 | 0.16 | 0.13 |
| 17. Do you notice imperfections in objects, like scratches on furniture, spots/stains, or frays on clothing, etc.? | 0.38 | -0.16 | -0.02 | 0.48 | 0.11 |
| 18. Do you prefer to finish one task before moving on to the next? | -0.02 | 0.45 | 0.00 | 0.04 | 0.48 |
| 27. Do you notice when pictures on walls are not lined up, or are crooked? | 0.36 | -0.10 | -0.03 | 0.51 | 0.07 |
| 28. Do you feel you have to complete a task once you have started it? | 0.00 | 0.49 | -0.01 | 0.11 | 0.44 |
| 33. Do you crack your joints (knuckles, neck, back, jaw, etc.)? | -0.04 | 0.01 | 0.30 | 0.07 | 0.19 |
| 39. Do you like to have a sense of evenness or balance, so if something touches one side of your body you have the urge to have it touch the other side of your body? | 0.05 | 0.07 | 0.26 | 0.12 | 0.25 |
| 45. Do you like to go to new places? | 0.03 | -0.72 | -0.07 | -0.01 | 0.66 |
| 53. Do you like to try new things? | 0.07 | -0.80 | 0.02 | 0.02 | 0.64 |
| Note. EFA = Exploratory Factor Analysis |  |  |  |  |  |

| **Supplementary Table 2** Item distribution of the Adult Routines Inventory subscales from the ESEM model | | | | |
| --- | --- | --- | --- | --- |
| **Factor** | **Description** | **Item** | **Factor loading** | **R square** |
| 1 | Insistence on sameness | 1. Do you prefer to do things in a particular order? | 0.91 | 0.82 |
|  |  | 2. Do you prefer to do things in a certain way? | 0.92 | 0.82 |
|  |  | 3. Are you attached to certain objects? | 0.32 | 0.34 |
|  |  | 6. Do you like to eat your meals in a certain order or certain way? | 0.41 | 0.46 |
|  |  | 7. Do you have persistent habits? | 0.69 | 0.67 |
|  |  | 9. Do you prefer to keep the same schedule or routine every day? | 0.75 | 0.63 |
|  |  | 19. Do you prepare for bedtime by engaging in a routine? | 0.50 | 0.41 |
|  |  | 37. Do you feel bothered if something disrupts your daily schedule or routine? | 0.64 | 0.64 |
|  |  | 50. Do you need to know when future events are happening so that you can plan for them? | 0.62 | 0.50 |
|  |  | 51. Once something is done a certain way do you feel like it has to be done that way every time? | 0.66 | 0.61 |
|  |  | 52. Do you insist that certain activities need to take place at a certain time? | 0.63 | 0.56 |
|  |  | 42. Do you order the same meals when you go to a particular restaurant? | 0.33 | 0.39 |
| 2 | Just right behaviours | 4. Are you concerned with dirt, cleanliness, or neatness? | 0.47 | 0.30 |
|  |  | 5. Do you arrange objects or perform certain behaviours until they are "just right?" | 0.71 | 0.70 |
|  |  | 8. Do you prefer to have the items on the top of your desks, tables, or counters lined up in straight lines, or in patterns? | 0.86 | 0.74 |
|  |  | 11. Do you prefer to have certain belongings "in their place?" | 0.55 | 0.58 |
|  |  | 22. Do things have to be "in their place" before you can get anything else done? | 0.65 | 0.69 |
|  |  | 36. Are you bothered when objects seem not to be lined up evenly? | 0.87 | 0.80 |
|  |  | 54. Do you clean or straighten up your home or office even when there may be more important things to do at that time? | 0.54 | 0.31 |
| 3 | Repetitive motor behaviours | 10. Do you fiddle with objects (tap pens, rip labels on bottles, etc.)? | 0.76 | 0.51 |
|  |  | 13. Do you twirl or play with your hair? | 0.57 | 0.32 |
|  |  | 16. Do you clench/grind your teeth during the day or when sleeping? | 0.42 | 0.24 |
|  |  | 21. Do you lick your lips until they are chapped? | 0.65 | 0.41 |
|  |  | 23. Do you chew non-edible objects (like pens or any other objects)? | 0.69 | 0.44 |
|  |  | 25. Do you fidget or bounce your legs when you are bored or anxious? | 0.55 | 0.42 |
|  |  | 31. Do you pick your skin? | 0.65 | 0.42 |
|  |  | 34. Do you feel the urge to clear your throat even when you don't have a cold or  allergies? | 0.32 | 0.28 |
|  |  | 35. Do you make odd sounds or noises? | 0.38 | 0.34 |
|  |  | 38. Do you tend to tap your fingers when you feel stressed or bored or are trying to concentrate? | 0.57 | 0.42 |
|  |  | 40. Do you bite your nails or skin on the fingers (cuticle)? | 0.60 | 0.30 |
|  |  | 41. Do you rock your body when you feel stressed, bored, or ill? | 0.49 | 0.42 |
|  |  | 43. Do you bite your lips/cheeks? | 0.69 | 0.50 |
|  |  | 44. Do you tug on your hair, eyelashes, or eyebrows? | 0.53 | 0.29 |
| 4 | Sensory sensitivity | 20. Do you avoid eating certain foods because of their "feel" or texture? | 0.36 | 0.44 |
|  |  | 24. Are you sensitive to loud noises? | 0.72 | 0.60 |
|  |  | 26. Are you sensitive to the ways that certain clothes feel? | 0.53 | 0.49 |
|  |  | 29. Does the sound of people eating bother you? | 0.46 | 0.30 |
|  |  | 30. Do you seem more aware of high-pitched noises than other people? | 0.79 | 0.69 |
|  |  | 32. Are you aware of buzzing or other sounds that come from lights or electronics? | 0.71 | 0.59 |
| 5 | Compulsions / Special interests | 46. Do you feel the urge to do or say things a certain number of times? | 0.42 | 0.35 |
|  |  | 47. Are you fascinated with one subject or activity? | 0.55 | 0.43 |
|  |  | 48. Do you recite lines from movies, videos, TV shows, or commercials that you have heard before? | 0.47 | 0.35 |
|  |  | 49. Are you good at imitating sounds or others' voices? | 0.53 | 0.31 |
|  |  | 55. Are there certain topics that, once you get started talking on them, it's difficult to stop? | 0.56 | 0.42 |
| Note. ESEM = Exploratory Structural Equation Modelling | | | | |
